# Effectiveness of a Swiss Ball Training Program on Quality of Life Among Women During the Climacteric Period

**DOI:** 10.7759/cureus.114895

**Published:** 2026-08-20

**Authors:** Tanvi B Bhujbal, Anandh S

**Affiliations:** 1 Department of Community Health Sciences, Krishna College of Physiotherapy, Krishna Vishwa Vidyapeeth (Deemed To Be University), Karad, IND

**Keywords:** climacteric period, menopause, pelvic floor muscle strength, quality of life, swissball training

## Abstract

Background

The climacteric period is a transitional stage in a woman’s life characterized by hormonal, physical, psychological, and functional measures that could negatively impact quality of life. Common symptoms include vasomotor disturbances, reduced muscle strength, decreased flexibility, impaired mobility, pelvic floor dysfunction, and psychological distress. Non-pharmacological interventions, particularly exercise-based programmes, have been progressively suggested for managing climacteric symptoms. Swiss ball training is a form of instability-based exercise that enhances core muscle activation, balance, flexibility, and neuromuscular coordination. Nevertheless, there is little information about its effectiveness in improving quality of life and functional outcomes among climacteric women. The aim to evaluate the efficiency of a Swiss ball training programme on quality of life among women during the climacteric period.

Methodology

This was a comparative study carried out in and around Karad throughout the span of an annual period. A total of 72 eligible women aged 40-60 years who had experienced three or more climacteric symptoms for at least six months were recruited using simple random sampling from the study population. After baseline assessment, participants were randomly allocated in a 1:1 ratio to either the Experimental Group (n = 36) or the Control Group (n = 36). The Oxford PERFECT score (OPS), Plank Hold Test (PHT), Hand-Held Dynamometer (HHD), Sit and Reach Test, Timed Up and Go Test (TUG), Menopause-Specific Quality of Life Questionnaire (MENQOL), and Greene Climacteric Scale (GCS) were used for baseline evaluations. For six weeks, the experimental group underwent a physiotherapist-supervised, structured Swiss ball training program three times a week, while the control group received standard care.

Results

The participants' average age was 50.15 ± 2.86 years, and all participants were in the climacteric phase. Baseline comparisons revealed that there were no notable distinctions between the two groups (p>0.05), indicating group homogeneity. Every outcome variable indicated statistically significant benefits in the experimental group after the intervention, according to within-group analysis. Significant improvements were observed in OPS (p=0.0121), PHT (p=0.0030), HHD right (p=0.0026), HHD left (p=0.0121), Sit and Reach Test (p=0.0452), TUG (p=0.0016), MENQOL (p=0.0001), and GCS (p=0.0221). Notable accomplishments were observed in MENQOL scores (t=6.808, p=0.0001), indicating substantial enhancement in menopause-specific quality of life. Between-group post-intervention analysis revealed significant differences favouring the experimental group in PHT (p=0.0479), HHD right (p=0.0493), Sit and Reach Test (p=0.0270), TUG (p=0.0468), and MENQOL (p=0.0471).

Conclusion

The findings of this comparative interventional study suggest that a structured Swiss ball training programme may improve quality of life, pelvic floor muscle strength, grip strength, flexibility, and functional mobility among women during the climacteric period. Swiss ball training may be considered a useful adjunct to physiotherapy management. However, the findings should be interpreted with caution due to the study design, relatively small sample size, and short intervention duration. Further large-scale randomized controlled trials with longer follow-up are needed to confirm its effectiveness and safety.

## Introduction

The climacteric period is a transitional stage in a woman’s life described by the gradual decline of ovarian function and the eventual cessation of menstruation. Numerous physiological, psychological, and social modifications that can have a considerable effect on general well-being and quality of life (QoL) are linked to this time. Hot flashes, nocturnal sweats, sleep difficulties, exhaustion, mood swings, anxiety, depression, and decreased physical functioning are frequent symptoms experienced by women during the climacteric period, all of which can impede everyday activities and social engagement [1].

Menopausal symptoms are extremely common throughout the climacteric period and may vary in severity among individuals. Vasomotor symptoms, emotional disturbances, and musculoskeletal complaints often contribute to reduced health status and decreased life satisfaction [2]. Beyond just causing physical discomfort, these symptoms can have a detrimental impact on psychological well-being, interpersonal relationships, and work performance, all of which can significantly lower one's QoL [3]. QoL is a multifaceted idea encompassing physical, psychological, social, and functional well-being. During the climacteric period, hormonal alterations may adversely influence these dimensions, leading to limitations in daily activities and decreased self-perceived health. Regular exercise has been recognized as an effective way to enhance QoL and promote overall well-being among women at this stage of life [4].

The decrease in estrogen levels during the climacteric period impacts multiple body systems, including the musculoskeletal system. Maintaining skeletal muscle mass, muscle strength, and functional performance all depend on estrogen. Reduced estrogen levels could contribute to muscle weakness, decreased physical capacity, and diminished functional independence [5]. These physiological changes often result in reduced participation in physical activities and may further accelerate the decline in physical fitness.

It has been illustrated that menopausal status and exercise levels are firmly correlated with physical performance. Compared to inactive women, women who continue to be physically active during the climacteric period exhibit superior functional performance and increased physical capacity [6]. As a result, exercise interventions have drawn more interest as non-pharmacological methods for improving QoL and controlling climacteric symptoms.

Regular exercise has been demonstrated to offer climacteric women many advantages. Exercise lessens the intensity of menopausal symptoms while also boosting cardiovascular fitness, muscular strength, flexibility, balance, and emotional well-being. These benefits support the integration of structured exercise programs exploring methods for promoting women's health experiencing climacteric changes [7].

Swiss ball training has become a well-known exercise modality that challenges stability and promotes activation of core musculature. The unstable surface of the Swiss ball requires continuous postural adjustments, enhancing neuromuscular coordination, balance, and back muscle engagement. This form of exercise has been widely utilized in fitness and rehabilitation settings due to its effectiveness in improving various components of physical fitness [8].

Core strengthening exercises carried out on a Swiss ball have demonstrated positive effects on muscular strength, endurance, flexibility, and balance among sedentary women. Enhanced core stability contributes to better posture, improved movement efficiency, and reduced risk of musculoskeletal discomfort [9]. These advantages might be especially helpful for women going through the climacteric phase, when their physical performance and muscle strength frequently decline.

Swiss ball workouts have been demonstrated to increase pelvic floor muscle function in addition to overall fitness. Because hormonal changes may affect the integrity and function of the pelvic floor, pelvic floor strengthening is particularly crucial during and after menopause. Gym ball training regimens have been linked to improvements in postmenopausal women's pelvic floor muscular strength [10].

Climacteric symptoms have a significant effect on women's QoL, affecting physical comfort, emotional stability, and social functioning. The cumulative effect of these symptoms often leads to decreased satisfaction with health and well-being. Consequently, interventions aimed at reducing symptom severity and improving physical function are vital for maintaining QoL during this transitional period [11].

Swiss ball exercises are also efficient in enhancing core stabilization and balance performance. Improved balance is particularly important for middle-aged and older women because declines in neuromuscular control and postural stability may increase the risk of falls and functional limitations. Enhanced core stability may support safer and more efficient movement during daily activities [12].

A crucial component of women's health throughout the climacteric phase is pelvic floor health. Preventing dysfunction and preserving QoL requires a thorough understanding and cognizance of pelvic floor function. The necessity for focused therapies and exercise regimens that address this crucial area is highlighted by the fact that many women have little knowledge of pelvic floor health [13].

Pelvic floor muscle function has been related to QoL and sexual well-being among peri- and postmenopausal women. Decreased pelvic floor muscle execution may contribute to discomfort, reduced sexual satisfaction, and impaired QoL. Interventions that improve pelvic floor function may therefore provide broad benefits extending beyond physical health alone [14].

Hormonal changes connected to estrogen deprivation may negatively affect pelvic floor muscle contractility and function. These alterations can increase susceptibility to pelvic floor disorders and contribute to reductions in functional well-being. Maintaining muscular strength and function through targeted exercise interventions may help counteract some of these effects [15].

The unstable surface of a Swiss ball escalates trunk muscle activation during exercise performance. Greater engagement of stabilizing muscles may enhance core strength and postural control, supporting improved functional capacity and movement efficiency. Such adaptations may be particularly advantageous for women experiencing age-related and hormonal changes affecting physical performance [16].

Muscle strength tends to decline during the menopausal transition, potentially affecting the capacity to perform daily activities independently. Reduced strength may be associated with lower levels of physical activity and decreased functional capacity. Strategies that promote muscular fitness are therefore essential for preserving health and independence during the climacteric period [17].

During the climacteric period, psychological symptoms like stress, worry, and emotional instability are frequent and can have a big impact on QoL. Therapeutic exercises and relaxation techniques have been demonstrated to be beneficial in fostering both physiological well-being and psychological relaxation. Exercise regimens that include these components may improve climacteric women's overall results [18].

Exercise involvement has additionally been demonstrated to positively influence mood among postmenopausal women. Improvements in emotional well-being, psychological health, and social functioning contribute substantially to enhanced QoL. Regular physical activity may therefore serve as a valuable non-pharmacological intervention to address both physical and psychological challenges associated with the climacteric period [19].

Swiss ball training was selected because it provides an unstable surface that promotes continuous activation of the core and pelvic floor muscles while improving balance, flexibility, coordination, and postural control. Unlike conventional floor-based exercises, Swiss ball exercises engage multiple muscle groups simultaneously and enhance neuromuscular control, which may help address the decline in muscle strength, balance, mobility, and quality of life commonly experienced during the climacteric period. Although various exercise interventions have been investigated for menopausal women, limited evidence is available regarding the effects of a structured Swiss ball training programme on both functional performance and menopause-related quality of life. Therefore, the present study aimed to address this gap by comparing a structured Swiss ball training programme with conventional exercise among climacteric women.

There is an increasing demand for safe, efficient, and easily available exercise therapies that can enhance physical fitness, pelvic floor function, psychological well-being, and general QoL in light of the complex difficulties encountered during the climacteric phase. By focusing on core stability, muscular strength, balance, flexibility, and functional performance, Swiss ball training provides a complete approach. There is, nevertheless, Minimal data that explicitly looks at its impact on women's QoL during the climacteric period. Thus, the goal of this research is to assess how well a Swiss ball training program affects women's QoL throughout the climacteric period.

We hypothesized that a structured Swiss ball training programme would be more effective than the conventional exercise programme in improving quality of life and functional performance among women during the climacteric period.

## Materials and methods

Study design

This study was conducted as a comparative interventional study to evaluate the effects of a structured Swiss ball training program on quality of life among women during the climacteric period. The study was carried out in and around Karad over one year. A total of 72 eligible participants were selected using simple random sampling and allocated equally into the experimental group (n = 36) and the control group (n = 36). Outcome measures were recorded before and after the six-week intervention period to compare the effects of the two exercise protocols. This study was conducted as a comparative interventional study to evaluate the effects of a structured Swiss ball training programme on quality of life and functional performance among women during the climacteric period.

Ethical considerations

Ethical approval for this study was obtained from the Institutional Ethics Committee of Krishna Vishwa Vidyapeeth (Deemed to be University), Karad (Approval No. 269/2025-2026). Written informed consent was obtained from all participants before enrolment, and the study was conducted in accordance with the principles of the Declaration of Helsinki.

Selection of participants

The inclusion and exclusion criteria of the participants are presented in Table 1.

**Table 1:** Inclusion and Exclusion Criteria for Participant Selection

Criteria	Inclusion	Exclusion
Age	Women aged 40 to 60 years	Women aged under 40 or over 60 years
Symptom Duration	Experiencing three or more climacteric symptoms for at least six months	Experiencing fewer than three symptoms or symptom duration of less than six months
Menstrual History	History of normal menstrual cycles	History of irregular, abnormal, or non-climacteric menstrual cycles
Hormonal Therapy	Free from active hormone replacement interventions	Currently undergoing hormone replacement therapy (HRT)
Physical & Medical Health	Status suitable for a structured rehabilitation or instability exercise program	Diagnosed neurological or musculoskeletal conditions; history of recent fractures, operations, or injuries that impair movement Co-occurring conditions comprising diabetes mellitus, hypertension, or cardiovascular diseases
Current Activity Level	Not engaged in external structured exercise protocols	Enrolled in any other organized physical fitness program
Compliance & Cognition	Willing to participate for the entire duration of the training program Capable of providing independent informed consent	Unwilling to commit to the study duration or exhibiting psychological disorders or cognitive limitations that make full engagement in the intervention impossible

Both Group A (interventional - Swiss ball) and Group B (conventional protocol) followed an identical overall framework of six weeks, three sessions per week at 45-60 minutes per session, structured across three progressive phases; however, they differ substantially in training modality, exercise selection, and emphasis. Group A uses the Swiss ball as the primary therapeutic tool throughout all phases, integrating it into balance, core, upper limb, lower limb, grip, and pelvic floor exercises, thereby creating an inherently unstable surface that simultaneously challenges proprioception, neuromuscular coordination, and postural control alongside conventional strength goals. Group B, by contrast, follows a more structured multi-component conventional approach with distinct emphasis on breathing exercises (diaphragmatic, thoracic expansion, and pursed-lip breathing, which are absent in Group A), progressive resistance using bands and dumbbells, and a more granular progression of grip strength using putty, towel wringing, and high-resistance tools. Both groups incorporate Kegel and pelvic floor activation, but Group B explicitly integrates Kegels with dynamic movements such as wall squats and glute bridges from Week 3 onward, potentially offering superior pelvic floor neuromuscular recruitment during functional tasks. Balance training in Group A progresses to eyes-closed variations on the ball by Weeks 5-6, targeting sensory integration and vestibular adaptation, whereas Group B progresses to foam pad standing and multi-directional clock-reach tasks, which more closely replicate real-world dynamic postural demands. Aerobic and dynamic components are comparatively more structured and progressive in Group B, incorporating stationary cycling and step aerobics by Phase 2, while Group A maintains simpler aerobic tasks such as brisk walking and treadmill use throughout. The cool-down in Group A evolves into box breathing, progressive muscle relaxation, and yoga-based postures by Weeks 5-6, offering a more mindfulness-oriented recovery component (Tables 2, 3).

**Table 2:** Detailed Weekly Protocol Comparison ROM- Range of motion TA- Transverse abdominus

Week / Phase	Group A – Interventional (Swiss Ball)	Group B – Conventional Protocol	Dosage
INITIAL PHASE			
Week 1–2 Initial Phase	Patient education & lifestyle counselling Warm-up • Shoulder shrugs, arm circles, neck rotation, high knees, ankle pump Aerobic • Brisk walking, seated marching (10 min) Balance (Swiss ball) • Tandem standing, single-leg stance, seated static on Swiss ball Upper limb • ROM, isometrics, dumbbell resistance Grip strengthening • Finger ROM, isometric finger press Lower limb • ROM, isometrics, ankle weight resistance Core & trunk • TA activation, gluteal bridge with hold Pelvic floor • Knack manoeuvre, Kegels, seated PF contractions on Swiss ball Flexibility • Free exercises, stretching Cool down • Diaphragmatic breathing, relaxation posture (5 min)	Patient education & lifestyle counselling Breathing exercises • Diaphragmatic breathing, thoracic expansion – 3 × 10 breaths Warm-up • Shoulder rotation, arm circles, neck rotation, leg swings, ankle pump Grip strengthening • Therapy ball squeeze, finger extension with band, pinch grip, wrist flexion/extension Balance • Tandem standing, single-leg stance, weight shifting, heel raises Upper limb • Wall push-ups, shoulder abduction with band, bicep curl, tricep wall press Lower limb • Mini squats, seated knee extension, hip abduction, calf raises Core strengthening • Pelvic tilt, Kegels, TA hold, gluteal bridge Dynamic (aerobic) • Brisk walk 10 min, marching in place 5 min Cool down • Ankle-toe movement, diaphragmatic breathing, calf/hamstring/quad stretches	Group A: 2 sets × 10 reps Group B: 2–3 sets × 10–15 reps Aerobic: 10 min Warm-up/Cool-down: 8–10 min
PROGRESSION PHASE 1			
Week 3–4 Progression Phase 1	Education: mindfulness, deep breathing, meditation Warm-up • + Leg swings added Aerobic • Treadmill/brisk walk, cycling (moderate resistance), stair climbing Balance (Swiss ball) • Tandem walk, seated weight shifts, hip marching on ball Upper limb (on ball) • Seated shoulder press, bicep curl, tricep extension on Swiss ball Grip • Ball squeeze in palm, tapping hands on ball, finger press on ball Lower limb (ball-assisted) • Wall squats with Swiss ball, seated leg extensions, glute bridge feet on ball, hamstring curl Core (ball) • TA activation on ball, alternate arm-leg extension, shoulder bridge, leg raise Pelvic floor • Seated PF contractions, bouncing on ball, pelvic tilts, marches with PF activation Flexibility • Ball-supported hamstring stretch, Swiss-ball bridge, seated spinal twist Cool down • Breathing, relaxation 2–3 min	Warm-up (same structure) Breathing • + Pursed-lip breathing added Grip (increased resistance) • Therapy ball squeeze (higher), putty pinch & roll, towel wringing, wrist band resistance Balance (progressed) • Tandem walk 3 m, single-leg stance without support, side-to-side stepping, heel-to-toe rocking Upper limb (resistance band) • Wall push-ups, lateral arm raises, bicep curl, overhead shoulder press, seated row Lower limb + Kegels • Wall squat + Kegels, glute bridge + Kegels, hip abduction with band, step-ups Core + Kegels • TA + Kegels, pelvic tilt, supine marching, dead bug (beginner) Dynamic • Brisk walk 10 min, forward/backward walk, stationary cycling Cool down • Triceps/biceps/pectoralis stretches, relaxation breathing	Group A: 3 sets × 10 reps Group B: 3 sets × 10–15 reps Aerobic: 10–15 min Balance: 10 min
PROGRESSION PHASE 2			
Week 5–6 Progression Phase 2	Education: stress-reduction continued Balance (eyes-closed variation added) • Tandem walk, seated weight shifts, hip marching on ball – eyes closed for static tasks Upper limb • Same as Wk 3–4 but increased to 12 reps Grip • Same exercises; increased to 12 reps Lower limb • + Ankle weights/theraband added; 12 reps Core (ball) • Same Swiss ball exercises; 12 reps Pelvic floor • Pelvic tilts combined with Kegels; 12 reps Flexibility • Full-body Swiss ball stretches (spinal twist, hamstring, back extension) Cool down • Box breathing (4-4-4-4), progressive muscle relaxation, yoga-based stretches (child’s pose, seated forward bend)	Grip (high resistance) • High-resistance ball squeeze, putty sculpt, wrist curls + reverse, farmer’s hold (3 × 30 sec) Balance (foam pad, clock reach) • Tandem walk 5 m, single-leg on foam pad, clock reach (5 directions), lateral crossover stepping Upper limb • Inclined push-ups, bent-over row, lateral + front raises, tricep kickback, external shoulder rotation Lower limb (full load) • Full squats, lunges, single-leg glute bridge, lateral band walk, step-ups (moderate height) Core (plank phase) • Forearm plank, wall sit, cat-and-camel, pelvic tilt hold, dead bug (full), TA hold + marching Dynamic • Forward/backward walk, stationary cycling, step aerobics Cool down • Full body stretches 20–30 sec each, relaxation breathing 5 min	Group A: 3 sets × 12 reps Group B: 3 sets × 12–15 reps Core holds: 20–30 sec Aerobic: 15–20 min
Week 8 – Follow-up assessment (both groups)			

**Table 3:** General Program Parameters

Duration	Frequency	Group A Modality	Group B Modality
6 weeks (both groups)	3 sessions/week · 45–60 min	Swiss ball-based training	Conventional resistance + balance

In summary, Group A protocol is well-suited to improving dynamic balance, neuromuscular coordination, and core stability through unstable surface training, while Group B protocol addresses a broader spectrum of physical fitness components including respiratory function, grip endurance, progressive resistance, and functional lower limb loading (Tables 2, 3). Post-intervention assessment was performed using the Oxford PERFECT score (OPS) [20], Plank Hold Test (PHT) [21], hand grip strength using a hand-held dynamometer (HHD) [22], Timed Up and Go Test (TUG) [23], Sit and Reach Test [24], Menopause-Specific Quality of Life Questionnaire (MENQOL) [25], and Greene Climacteric Scale (GCS) [26].

Statistical analysis

Data were analyzed using SPSS version 26.0 (IBM Corp., Armonk, NY, USA). Normality of the data was assessed using the Shapiro-Wilk test. Continuous variables are presented as mean ± standard deviation. For normally distributed data, parametric tests were applied. Paired t-tests were used for within-group comparisons, and independent t-tests were used for between-group comparisons. A p-value of <0.05 was considered statistically significant.

Outcome measures

Oxford PERFECT Score

The OPS was used to assess pelvic floor muscle strength and function. The OPS was used to determine pelvic floor muscle strength and function. It is a reliable and valid clinical assessment tool with reported inter-rater reliability ranging from intraclass correlation coefficient (ICC) = 0.72-0.93 and intra-rater reliability ranging from ICC = 0.81-0.95. The scale also illustrates good concurrent validity with objective measures such as perineometry and electromyography (r = 0.70-0.90). Pelvic floor muscle strength was evaluated through vaginal digital palpation and graded using the Oxford scale along with the PERFECT components. This scale is an open-access scale [20]. 

Plank Hold Test

The PHT was utilized to find core muscle endurance. With ICC values between 0.95 and 0.99, it exhibits outstanding test-retest reliability. demonstrates good criterion validity, showing significant correlations with other measures of core muscle endurance (r = 0.71-0.89). Participants were instructed to maintain a standard forearm plank position, and the maximum duration for which the position could be held correctly was recorded in seconds [21].

Handheld Dynamometer

Hand grip strength was quantified using a HHD to assess overall muscle strength. This method has excellent test-retest reliability, with ICC values ranging from 0.95 to 0.98, and strong criterion validity with overall muscular strength and functional performance measures (r = 0.80-0.90). Participants were instructed to perform a maximal grip contraction, and the highest value obtained was recorded in kilograms [22].

Timed Up and Go Test

The TUG test was used to examine functional mobility and dynamic balance. It is a highly reliable measure, with intra-rater and inter-rater reliability values ranging from ICC = 0.95-0.99. The test has demonstrated good concurrent validity with other balance and mobility assessments (r = 0.61-0.81). Participants were asked to stand up from a chair, walk 3 metres, turn around, return, and sit down while the total time taken was recorded in seconds [23].

Sit and Reach Test

The Sit and Reach Test was used to evaluate the flexibility of the hamstring and lower back muscles. The test has high test-retest reliability, with ICC values ranging from 0.89 to 0.98, and moderate to strong validity for measuring hamstring flexibility (r = 0.71-0.89). Participants sat with their legs extended and reached forward as far as possible, and the maximum distance reached was recorded in centimetres [24].

Menopause-Specific Quality of Life Questionnaire

The MENQOL was employed to ascertain the impact of menopausal symptoms on quality of life. The questionnaire has excellent internal consistency, with Cronbach's alpha values ranging from 0.81 to 0.95, and good construct validity demonstrated through correlations with menopausal symptom measures (r = 0.60-0.88). It consists of 29 items covering vasomotor, psychosocial, physical, and sexual domains [25]. 

Greene Climacteric Scale

The GCS was employed to examine the severity of climacteric symptoms. The scale demonstrates high internal consistency, with Cronbach's alpha values ranging from 0.83 to 0.87, and good construct validity with menopausal symptom severity and QoL measures (r = 0.65-0.85). The questionnaire includes psychological, somatic, vasomotor, and sexual symptom domains, with higher scores indicating greater symptom severity [26]. 

Procedure

Participants who met the eligibility criteria and provided written informed consent underwent baseline assessment. They were then randomly allocated into the experimental group (Swiss ball training) and the control group (conventional exercise) using a computer-generated randomization sequence with a 1:1 allocation ratio. Allocation concealment was maintained using sequentially numbered, opaque, sealed envelopes opened after participant enrollment. Owing to the nature of the intervention, participant and therapist blinding was not feasible; however, outcome assessment and statistical analysis were performed by investigators blinded to group allocation.

## Results

The descriptive characteristics of the 72 participants featured in the study. The mean age of the participants was 50.15 ± 2.86 years, and 100% of the participants (n = 72) were confirmed to be in the climacteric phase. The baseline outcome measures showed mean scores of 3.44 ± 0.49 for OPS, 26.11 ± 10.80 for PHT, 18.40 ± 3.87 for right HHD, and 17.86 ± 3.66 for left HHD. The mean values for Sit and Reach, TUG, MENQOL, and GCS were 50.15 ± 2.86, 16.04 ± 4.17, 90.37 ± 7.04, and 49.97 ± 3.57, respectively. Overall, the findings show that the study participants had relatively homogeneous baseline characteristics, providing a suitable sample for evaluating the intervention outcomes, as given in Table 4.

**Table 4:** Descriptive statistics of overall participants (n= 72) OPS- Oxford PERFECT Scale [20], PHT- Plank Hold Test [21], HHD- Hand Held Dynamometer [22], TUG- Time Up and Go test [24], MENQOL- Menopause Specific Quality of Life Scale [25], GCS- Greene Climacteric Scale [26]

Variables	Mean (SD) or Frequency(%)
Age, mean(SD)	50.15±2.86
Climacteric Phase, n%	
Yes	72(100)
OPS	3.44±0.49
PHT	26.11±10.80
HHD (right)	18.40±3.87
HHD (left)	17.86±3.66
Sit and Reach Test	50.15±2.86
TUG	16.04±4.17
MENQOL	90.37±7.04
GCS	49.97±3.57

The baseline (pretest) qualities of the control and experimental groups before the intervention. The results display that there were no statistically substantial similarities between the two groups in age, OPS, PHT, HHD (right and left), Sit and Reach, TUG, MENQOL, and GCS scores, as all p-values were greater than 0.05. Additionally, all participants in both groups were in the climacteric phase (100%). These findings indicate that the control and experimental groups were comparable at baseline with respect to demographic and outcome variables. Since no statistically significant differences were observed in the baseline characteristics between the two groups, the post-intervention improvements are more likely to reflect the effects of the respective exercise interventions. However, other factors such as participant adherence, lifestyle changes, and expectation effects may also have influenced the outcomes and should be considered when interpreting the findings, as given in Table 5.

**Table 5:** Baseline characteristics of the study participants. The Chi-square test was used to compare the categorical variable (Climacteric Phase), while the independent t-test was used to compare continuous variables following confirmation of normality using the Shapiro-Wilk test. OPS- Oxford PERFECT Scale [20], PHT- Plank Hold Test [21], HHD- Hand Held Dynamometer [22], TUG- Time Up and Go test [24], MENQOL- Menopause Specific Quality of Life Scale [25], GCS- Greene Climacteric Scale [26]

Variables	Control (n=36)	Experimental (n= 36)	T value	P value
Age, mean (SD)	50.6±3.01	49.63±2.59	1.528	0.1311
Climacteric Phase, n%				
Yes	36	36		
OPS	3.41±0.49	3.47±0.49	0.4684	0.6409
PHT	26.0±12.6	26.16±8.8	0.04301	0.9658
HHD (right)	18.1±3.77	18.63±3.95	2.5113	2.6108
HHD (left)	17.5±3.52	18.16±3.77	0.7002	0.4862
Sit and Reach Test	-10.1±5.32	19.19±3.69	0.04325	0.9656
TUG	16.47±4.43	15.6±3.84	0.8682	0.3883
MENQOL	90.33±7.40	90.4±6.67	0.04947	0.9607
GCS	50.41±3.93	49.52±3.11	1.047	0.2986

The within-group comparison of pretest and post-test outcome measures in the control group. Significant enhancements were observed in OPS (p = 0.0318), HHD (left) (p = 0.0231), Sit and Reach (p = 0.0103), TUG (p = 0.0438), MENQOL (p = 0.0385), and GCS (p = 0.0209), indicating positive changes over the study period. However, there were no statistically significant variations discovered in PHT (p = 0.2814) and HHD (right) (p = 0.6286), indicating the persistence of these variables relatively unchanged. The mean differences between pretest and post-test scores were generally small, reflecting modest improvements in functional performance, flexibility, quality of life, and symptom-related outcomes. In general, the conclusions show that there were significant variations in the control group in several outcome measures, although the magnitude of improvement was limited, as given in Table 6.

**Table 6:** Within the control group comparison of pre-test and post-test data of the outcome measures OPS- Oxford PERFECT Scale [20], PHT- Plank Hold Test [21], HHD- Hand Held Dynamometer [22], TUG- Time Up and Go test [24], MENQOL- Menopause Specific Quality of Life Scale [25], GCS- Greene Climacteric Scale [26]

Variables	Pretest	Post-test	Mean difference	t value	P value
OPS	3.41±0.49	3.58±0.68	0.17	2.236	0.0318
PHT	26.0±12.6	26.19±12.6	0.19	1.094	0.2814
HHD (right)	18.1±3.77	18.36±3.62	0.26	0.4880	0.6286
HHD (left)	17.5±3.52	17.69±3.58	0.19	2.376	0.0231
Sit and Reach Test	-10.1±5.32	-9.8±5.30	0.30	2.712	0.0103
TUG	16.47±4.43	16.36±4.60	-0.11	2.092	0.0438
MENQOL	90.33±7.40	90.91±7.83	0.58	2.150	0.0385
GCS	50.41±3.93	50.02±4.28	-0.39	2.490	0.0209

The within-group comparison of pretest and post-test outcome measures within the group under experimentation. Profits that are statistically significant were seen in all measured variables, including OPS, PHT, HHD (right and left), Sit and Reach, TUG, MENQOL, and GCS, as all p-values were less than 0.05. The greatest improvement was noted in MENQOL (t = 6.808, p = 0.0001), indicating a marked enhancement in menopause-related quality of life following the intervention. Significant improvements were also seen in muscle strength, flexibility, functional mobility, and overall health-related outcomes, as reflected by improvements in HHD, Sit and Reach, TUG, and GCS scores. All things taken into account, these results show that the intervention was successful in bringing about notable improvements in every end measure for the experimental group, as given in Table 7.

**Table 7:** Within the experimental group comparison of pre-test and post-test data of the outcome measures OPS- Oxford PERFECT Scale [20], PHT- Plank Hold Test [21], HHD- Hand Held Dynamometer [22], TUG- Time Up and Go test [24], MENQOL- Menopause Specific Quality of Life Scale [25], GCS- Greene Climacteric Scale [26]

Variables	Pretest	Post-test	Mean difference	t	P value
OPS	3.47±0.49	3.63±0.58	00.16	2.646	0.0121
PHT	26.16±8.8	26.66±8.84	0.50	3.188	0.0030
HHD (right)	18.63±3.95	19.19±3.69	0.56	3.247	0.0026
HHD (left)	18.16±3.77	18.36±3.62	0.20	2.646	0.0121
Sit and Reach Test	-10.19±3.69	-8.63±6.18	-27.82	2.877	0.0452
TUG	15.6±3.84	15.11±4.42	-0.49	3.416	0.0016
MENQOL	90.4±6.67	91.22±7.23	0.82	6.808	0.0001
GCS	49.52±3.11	48.86±3.69	0.66	2.395	0.0221

The post-intervention comparison between the control and experimental groups across the outcome measures. In addition to statistical significance, the clinical relevance of the observed improvements should be considered. The experimental group demonstrated improvements in functional performance and menopause-related quality of life that are likely to be clinically meaningful. However, established minimal clinically important difference (MCID) values are not available for all outcome measures used in this study. Therefore, while the findings suggest potential clinical benefits of Swiss ball training, further studies incorporating validated MCID thresholds and patient-reported outcome measures are required to better determine the clinical significance of these improvements. The experimental group showed more improvement than the control group in PHT (p = 0.0479), HHD (right) (p = 0.0493), Sit and Reach (p = 0.0270), TUG (p = 0.0468), and MENQOL (p = 0.0471). However, no notable distinctions were discovered for OPS (p = 0.7156), HHD (left) (p = 0.4628), and GCS (p = 0.2086), suggesting comparable outcomes between the groups for these measures. The experimental group generally demonstrated better post-intervention performance, particularly in muscle strength, flexibility, functional mobility, and menopause-related quality of life. When compared to the control condition, the results indicate that the intervention was successful in achieving better results in a number of important health and functional criteria, as given in Table 8.

**Table 8:** Comparison of post-intervention values of OPS, PHT, HHD, Sit and Reach Test, TUG, MENQOL and GCS and BMI between control group (n=36) and experimental group (n=36) OPS- Oxford PERFECT Scale [20], PHT- Plank Hold Test [21], HHD- Hand Held Dynamometer [22], TUG- Time Up and Go test [24], MENQOL- Menopause Specific Quality of Life Scale [25], GCS- Greene Climacteric Scale [26]

Variables	Post-intervention mean		Mean difference	t	p
	Control	Experimental			
OPS	3.58±0.68	3.63±0.58	0.05	0.3658	0.7156
PHT	26.19±12.6	26.66±8.84	0.53	2.014	0.0479
HHD (right)	18.36±3.62	19.19±3.69	0.83	2.001	0.0493
HHD (left)	17.69±3.58	18.36±3.62	0.67	0.7383	0.4628
Sit and Reach Test	-9.8±5.30	-8.63±6.18	1.17	2.309	0.0270
TUG	16.36±4.60	15.11±4.42	-1.25	2.061	0.0468
MENQOL	90.91±7.83	91.22±7.23	0.31	2.058	0.0471
GCS	50.02±4.28	48.86±3.69	-1.16	1.281	0.2086

## Discussion

The present study analyzed the effects of a structured Swiss ball training programme on QoL, grip strength, pelvic floor muscle function, flexibility, and functional mobility in climacteric women (mean age 50.15 ± 2.86 years). All 72 participants (n = 36 control, n = 36 experimental) were confirmed to be in the climacteric phase, and the groups were statistically comparable at baseline across all measured variables (p > 0.05). The comparable baseline characteristics between the groups reduced the likelihood that pre-existing differences influenced the outcomes. Therefore, the observed post-intervention differences are more likely to reflect the comparative effects of the two exercise interventions, although they cannot be attributed solely to the Swiss ball training programme.

The MENQOL score showed the most notable improvement (t = 6.808, p = 0.0001), indicating a clinically significant improvement in menopause-related QoL. Within-group analysis of the experimental group demonstrated benefits that were statistically significant across all eight outcome measures post-intervention (p < 0.05). Between-group post-intervention analysis demonstrated statistically significant differences in pelvic floor muscle strength measured by perineometry (PHT; p = 0.0030) and right-hand grip strength measured using a hydraulic hand dynamometer (HHD right; p = 0.0493), indicating greater improvements in the experimental group compared with the control group. Furthermore, functional mobility via the TUG (p = 0.0016) demonstrated significant within-group improvement.

Between-group post-intervention comparisons confirmed that the experimental group significantly surpassed the control group in PHT (p = 0.0479), HHD right (p = 0.0493), Sit and Reach (p = 0.0270), TUG (p = 0.0468), and MENQOL (p = 0.0471), reinforcing the efficacy of the Swiss ball programme as a multimodal rehabilitative intervention for climacteric women. The GCS and the OPS demonstrated significant within-group changes in the experimental group (GCS: p = 0.0221; OPS: p = 0.0121), but the between-group comparisons for these measures failed to reach statistical significance (GCS: p = 0.2086; OPS: p = 0.7156), indicating that prolonged training sessions could be necessary to differentiate at the group level.

The enhancements seen throughout this study’s outcome domains are physiologically coherent and mechanistically grounded in the endocrine and neuromuscular changes characteristic of the climacteric period. The fundamental driver of climacteric symptom burden is the progressive depletion of ovarian follicular reserve, resulting in a sustained decline in oestradiol, progesterone, and inhibin B. Following natural menopause, ovarian estrogen production declines, resulting in a compensatory increase in circulating gonadotropins, particularly follicle-stimulating hormone (FSH) and luteinizing hormone (LH). Serum FSH concentrations during natural postmenopause are typically reported to be approximately 10-20 times higher than premenopausal levels, reflecting average physiological changes associated with ovarian follicular depletion rather than peak values. This hormonal alteration is a well-recognized endocrine feature of the menopausal transition. The resulting hypoestrogenic state hinders skeletal muscle protein production by negatively affecting the oestrogen receptor-alpha-mediated suppression of the ubiquitin-proteasome proteolytic pathway, disrupts thermoregulatory control via the hypothalamic thermostat, attenuates serotonergic and GABAergic neurotransmission, and speeds up bone resorption through disinhibited osteoclast activity [27].

The present study similarly observed highly significant MENQOL improvement (t = 6.808, p = 0.0001), suggesting that the multi-dimensional, physically engaging nature of Swiss ball training produces psychological benefits comparable to those reported for aerobic and mind-body modalities. The present investigation did not necessarily isolate vasomotor symptom frequency as an independent endpoint; however, the overall increase in MENQOL encompasses the vasomotor domain, indicating simultaneous positive effects on this symptom cluster. The current investigation supports these conclusions in a specifically climacteric population, demonstrating analogous gains in flexibility (Sit and Reach improvement: 1.56 cm, p = 0.0452) and functional mobility (TUG: p = 0.0016), while extending the evidence base by documenting improvements in MENQOL, an outcome not examined by Sekendiz et al. [9].

The present study’s significant right-hand HHD improvement (mean difference 0.56 kg, p = 0.0026) in the experimental group challenges the notion that grip strength decline during the climacteric is inevitable, demonstrating that the grip-intensive stabilising demands of Swiss ball upper limb exercises can provide meaningful anabolic stimulus to the upper extremity musculature sufficient to produce measurable strength gains within the intervention timeframe.

The significant TUG improvement in the experimental group (mean reduction 0.49 seconds, p = 0.0016) carries direct clinical relevance to fall prevention in postmenopausal women. Behm et al. (2010) [27] demonstrated that instability-based training using a Swiss ball engages proprioceptive and neuromuscular control systems more comprehensively than stable-surface training, with particular benefits for coordination and postural stability. The TUG outcome data of the current study provide applied, real-world confirmation of this laboratory neuromuscular conclusion in a clinically relevant postmenopausal population.

The significant PHT improvement (mean difference 0.50 units, p = 0.0030) in the experimental group represents, to the best of the authors’ knowledge, the first controlled evidence that Swiss ball training produces measurable pelvic floor strength gains in climacteric women without requiring isolated pelvic floor exercises.

The MENQOL improvement recorded in the experimental group (t = 6.808, p = 0.0001) represents the strongest statistical finding of the entire study. The MENQOL captures QoL across the four domains of vasomotor, psychosocial, physical, and sexual functioning that are most directly impacted by the climacteric transition. The GCS data showed significant within-group improvement in the experimental group (p = 0.0221) but did not distinguish significantly between groups at post-intervention (p = 0.2086) [26]. This pattern could represent the GCS’s broader and more symptom-oriented scope relative to the MENQOL, capturing psychological, somatic, and vasomotor symptoms that may require a longer intervention period or higher training intensity to show group-level differentiation. The within-group GCS improvement nonetheless supports the conclusion that the Swiss ball program produced genuine reductions in overall climacteric symptom burden in the experimental group.

Khan et al. (2013) demonstrated that psychological factors such as depression and anxiety can influence the outcomes of pelvic floor muscle training in individuals with pelvic floor dysfunction. Their findings suggested that patients with greater psychological distress may have poorer responses to pelvic floor muscle training, highlighting the importance of considering psychological status alongside physical rehabilitation. These findings are similar to the existing study, as they emphasize that factors beyond the physical intervention can influence rehabilitation outcomes and functional improvement [28].

Escamilla et al. (2010) demonstrated that core muscle activation varies between Swiss-ball and traditional abdominal exercises, with both exercise approaches producing activation of the abdominal musculature, although the level of activation differed according to the exercise performed [29]. Sipilä et al. (2020) further demonstrated that muscle mass and physical activity are associated with muscle and bone characteristics in middle-aged women, with menopausal status also influencing these outcomes [30]. Similarly, Wilk et al. (2010) reported findings regarding core muscle activation during Swiss-ball and traditional abdominal exercises, supporting the importance of exercise selection when targeting core musculature [31]. These findings are in agreement with the existing study, as they collectively support the role of targeted physical activity and exercise in improving or maintaining muscular function and physical capacity.

Consistent with Jurgensen et al. (2010) [32], improvements in fitness may positively influence pelvic floor function. Berin et al. (2019) [33] further demonstrated that structured resistance training significantly reduced hot flush severity in postmenopausal women. Marshall and Murphy (2005) [34] showed that Swiss ball exercises enhance core muscle activation through instability-based training. Additionally, the dynamic and engaging nature of Swiss ball training may improve motivation and adherence compared to conventional exercise programmes [32,35].

The present study demonstrates significant increases in Sit and Reach scores in the experimental group following the Swiss ball training programme (p = 0.0452), with post-intervention values also showing a significant advantage over the control group (p = 0.0270). These results are robust, aligning with Sekendiz et al. (2010) [9], who reported that Swiss ball-based exercises significantly enhance flexibility through dynamic stretching, improved muscle activation, and increased range of motion. The instability provided by the Swiss ball promotes continuous engagement of core and postural muscles, thereby improving overall flexibility and functional movement. Similarly, OPS scores showed significant within-group improvement in the experimental group (p = 0.0121), although no significant difference was observed between groups post-intervention (p = 0.7156). Enhanced neuromuscular coordination, pelvic stability, and physical conditioning from Swiss ball training-which has been demonstrated to boost core muscle activation and trunk stability-may be responsible for the improvement in OPS [9]. These results imply that Swiss ball exercises can enhance physical functioning and flexibility, improving general health throughout the climacteric phase.

The present study has certain limitations. The short intervention duration limits conclusions regarding long-term effects on quality of life and bone health. Outcomes were primarily based on self-reported measures, which may be influenced by response bias. The research was carried out in a single setting, limiting generalisability to broader populations. Mechanistic interpretation was limited because hormonal biomarkers and bone mineral density were not evaluated. Furthermore, it is impossible to assess the sustainability of the noted advantages in the absence of long-term follow-up.

Future studies should evaluate the long-term effects of Swiss ball training on QoL, body composition, and bone health in climacteric women. Larger multicentre trials, objective assessments such as Dual-Energy X-ray Absorptiometry (DEXA) and hormonal markers, and comparisons with other exercise modalities are recommended. Combining physical and psychological interventions may further enhance outcomes.

The present study compared Swiss ball training with an active conventional exercise programme rather than a no-treatment control. Therefore, the between-group differences should be interpreted as the comparative effects of two exercise interventions and may be influenced by differences in exercise type, intensity, and neuromuscular demands. Although greater improvements were observed in the Swiss ball training group, these findings do not imply that the benefits were solely attributable to Swiss ball training.

Limitation

A limitation of this study is that the control group received an active conventional exercise programme rather than a no-treatment control. Consequently, the observed differences between groups may reflect variations in exercise type or intensity in addition to the specific effects of Swiss ball training. The study evaluated multiple outcome measures, which may have increased the risk of Type I error. In addition, effect sizes and confidence intervals were not reported, limiting the interpretation of the magnitude and precision of the observed effects.

Strengths 

The present study compared a structured Swiss ball training programme with an active conventional exercise programme using standardized intervention protocols and validated outcome measures. The findings provide clinically relevant evidence on the comparative effects of these two physiotherapy interventions for improving functional performance and quality of life among women during the climacteric period. The study also employed a supervised exercise programme with good participant adherence, enhancing the clinical applicability of the findings.

Future scope

Future studies should include larger sample sizes, multicentre populations, and longer follow-up periods to evaluate the long-term effects of Swiss ball training among climacteric women. Randomized controlled trials comparing Swiss ball training with different physiotherapy interventions and varying exercise intensities are needed to establish its comparative effectiveness and safety. Further research should also investigate adherence, cost-effectiveness, and the underlying mechanisms responsible for improvements in functional performance and QoL.

## Conclusions

The current study concludes that a structured Swiss ball training program is an effective, safe, and non-pharmacological intervention for improving QoL and functional performance among climacteric women. Significant improvements were observed in pelvic floor muscle strength, grip strength, flexibility, mobility, and QoL. Swiss ball training may improve selected functional and QoL outcomes among women during the climacteric period and may be considered a potential adjunct to physiotherapy-based management. However, further well-designed randomized controlled trials with larger sample sizes and longer follow-up are required before recommending its routine clinical implementation. Further large-scale, longer-term intervention studies and follow-up assessments are recommended to establish long-term effectiveness and optimize training protocols.
